# Using Social Networking to Understand Social Networks: Analysis of a Mobile Phone Closed User Group Used by a Ghanaian Health Team

**DOI:** 10.2196/jmir.2332

**Published:** 2013-04-03

**Authors:** Nadi Nina Kaonga, Alain Labrique, Patricia Mechael, Eric Akosah, Seth Ohemeng-Dapaah, Joseph Sakyi Baah, Richmond Kodie, Andrew S Kanter, Orin Levine

**Affiliations:** ^1^Global Disease Epidemiology and ControlInternational Health DepartmentJohns Hopkins Bloomberg School of Public HealthBaltimore, MDUnited States; ^2^Earth InstituteCenter on Globalization and Sustainable DevelopmentColumbia UniversityNew York, NYUnited States; ^3^mHealth AllianceUnited Nations FoundationWashington, DCUnited States; ^4^BonsaasoGhanaMillennium Villages ProjectManso-NkwantaGhana

**Keywords:** mobile health, electronic health, telehealth, sociology, social network analysis, rural health, global health, evaluation research, Ghana

## Abstract

**Background:**

The network structure of an organization influences how well or poorly an organization communicates and manages its resources. In the Millennium Villages Project site in Bonsaaso, Ghana, a mobile phone closed user group has been introduced for use by the Bonsaaso Millennium Villages Project Health Team and other key individuals. No assessment on the benefits or barriers of the use of the closed user group had been carried out.

**Objective:**

The purpose of this research was to make the case for the use of social network analysis methods to be applied in health systems research—specifically related to mobile health.

**Methods:**

This study used mobile phone voice records of, conducted interviews with, and reviewed call journals kept by a mobile phone closed user group consisting of the Bonsaaso Millennium Villages Project Health Team. Social network analysis methodology complemented by a qualitative component was used. Monthly voice data of the closed user group from Airtel Bharti Ghana were analyzed using UCINET and visual depictions of the network were created using NetDraw. Interviews and call journals kept by informants were analyzed using NVivo.

**Results:**

The methodology was successful in helping identify effective organizational structure. Members of the Health Management Team were the more central players in the network, rather than the Community Health Nurses (who might have been expected to be central).

**Conclusions:**

Social network analysis methodology can be used to determine the most productive structure for an organization or team, identify gaps in communication, identify key actors with greatest influence, and more. In conclusion, this methodology can be a useful analytical tool, especially in the context of mobile health, health services, and operational and managerial research.

## Introduction

The Millennium Villages Project (MVP) is a collaborative initiative that seeks to support and empower rural African communities to achieve the Millennium Development Goals by 2015. Initiatives are centered on five sectors: agriculture, health, education, enterprise, and infrastructure. Within the health sector, science and technologies are being integrated into health systems and are evaluated in close partnership with the communities and other key stakeholders (ie, Ministries of Health) in each of the 14 MVP sites.

In the MVP site in Bonsaaso, Ghana, a mobile phone closed user group (CUG) has been introduced for use by the Bonsaaso MVP Health Team and other key individuals representing the Ghana Ministry of Health and Ghana Health Service at a local level. Cited benefits of mobile phone CUG include the following [1-4]:

- Improved management and savings: the ability for the organization to more effectively manage communication costs among staff (and potential savings).
- Limitless network size: flexibility in the number of members that can be added to the group (the closed network can scale up or down).
- Increased intra-network access: members of the group can call one another, at any time, in an unlimited capacity.

In part, it was these benefits that prompted the establishment of a mobile phone CUG in each of the MVP sites for the health teams. The CUG has been in place in Bonsaaso since 2009. No assessment of the CUG has been carried out. Consequently, it was not known if and how beneficial the closed user group is to the health staff and their clients. Based on the organizational structure of the Bonsaaso MVP Health Team, it was inferred that aspects of the traditional social network would be mirrored in the social network resulting from the introduction of the mobile phone CUG. Therefore, it was hypothesized that Community Health Nurses would be the most central nodes (or are central actors) in the closed user group network as they serve as intermediaries in the command structure. To determine the validity of this hypothesis, social network analysis methods were used to explore the “social networks” within the mobile phone CUG. By using social network analysis methods, it was believed that the evaluation of the relational ties would allow for more in-depth analysis of the social context in which the closed user group was introduced, potentially changed, and now operates.

For the purposes of this paper, mobile health (mHealth) has been defined in accordance with the definition found in the World Health Organization’s Global Observatory for eHealth 2011 Survey as “medical and public health practice supported by mobile devices, such as mobile phones…” [5]. Experts in mHealth have noted that there is not only a need for “high-quality research” in mHealth, but traditional public health methods, such as randomized controlled trials, may not be possible for all mHealth project evaluations [6]. This applies to the context of the MVP closed user groups where randomized controlled trials would require extensive resources and a highly complex study design and analysis.

Douglas Luke underlined the need to move beyond traditional public health methods and look at methods that would allow for more contextual information in community-based research [7]. Social network analysis is a new tool for public health that can be used to explore and answer questions that were not answerable by previous methods [7,8]. For example, this tool can be used to provide information on social and environmental contexts, better explain or provide structure to abstract concepts through mapping and visualization, and present new or previously known information in unique ways for different and additional insights [9-11].

Social network analysis can be used as a managerial tool as well [12-16]. Those managing organizations can maximize their network and employee performance by understanding the informal network. They can gain insight on advice, trust, and communication, in addition to identifying the influential players, gaps in the information flow, and inefficient use of resources [13,17]. This information can then be used to develop appropriate interventions [16].

Important attributes of the [informal] networks that should be assessed to better understand the network include centrality, degree, and betweenness [14,18]. Balkundi and Harrison found that the more dense a network, the better the performance and viability of the team [19]. Granovetter and Burt, separately, suggest that weak ties should also be assessed. Weak ties provide a larger picture of the network and may also explain the formation of subgroups or cliques within a network [20]. They may also provide insight into who may have social capital within a network (eg, those who broker information across the weak ties and, in effect, prevent disconnection in the network) and potentially help explain performance in the network [21,22]. All of these measures were taken into consideration for the assessment of the Bonsaaso MVP CUG.

## Methods

This research was part of a larger evaluation study, the Millennium Villages Project and OASIS II Research on MVG-Net, approved by Columbia University (IRB-AAAF1647).

**Figure 1:**
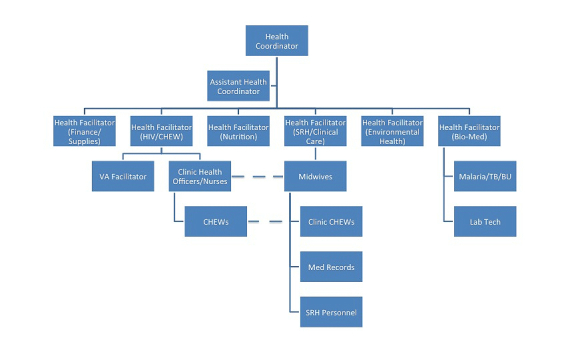
Traditional organogram of the MVP Bonsaaso Health Team.

Five sectors are represented at the Bonsaaso MVP site. The teams (or sectors) are agriculture, infrastructure, enterprise, education, and health. Coordinators lead each of the five sectors and report to the Team Leader, who also functions as the Science Coordinator. The health team’s traditional organogram depicts the non-CUG flow of information, highlighting the siloed and rigorous chain of command in the structure (see Figure 1). The Health Coordinator leads the Health Team. Two Health Facilitators are designated to manage the community-based Health Team. The community-based Health Team includes midwives, Community Health Nurses, and Community Health Extension Workers. One facilitator oversees Community Health Nurses and Community Health Nurses, in turn, oversee Community Health Extension Workers. A separate Health Facilitator oversees midwives. Midwives run the seven clinics in Bonsaaso and work with both the Community Health Nurses and clinic-based Community Health Extension Workers, who are distributed across the 30 communities, or villages. Based on the traditional hierarchy, it appears that Community Health Nurses relay information not only between Community Health Extension Workers and midwives, but also between Community Health Extension Workers and Health Team management members.

Figure 1 shows a traditional organogram of the MVP Bonsaaso Health Team. This organogram is not inclusive of the two ambulance drivers. While they are not included in the organogram, they are considered to be members of the Health Team. They typically report to the Health Coordinator but work closely with the midwives.

Officially, the mobile phone CUG in MVP consists of 79 members. This includes the midwives, Community Health Nurses, Community Health Extension Workers, Health Facilitators, Health Coordinator, Team Leader, physicians at the local referral hospital, ambulance drivers, and local government representatives.

As this network involved a single set of actors and their relations, subsequent analyses were based on one-mode networks. The type of relational tie being assessed was nondirectional communication, or exchange of information, among the actors in the network, as the existence of communication (or a tie) was the most important factor.

The datasets for the social network analyses were based on monthly voice data from Airtel Bharti Ghana. The voice data were formatted in an excel matrix that had the numbers of those in the CUG as the top row and left-most column. Initially, the relational data were provided as the total duration of calls made between two actors over the time period of interest in minutes. This information was converted to binary form as the interest was primarily in the establishment of communication among the actors in the network within a 1-month time period, rather than the duration of the communication. If no communication took place between 2 actors, this was represented in the matrix as “0”. If communication did take place between 2 actors, this was represented as “1” in the dataset. The available datasets represent each of the months from March 2011 through September 2011. A facsimile of the traditional network was also used to illustrate the foundation of the hypothesis. The data were not initially in symmetric form, therefore, the primary researcher reformatted the datasets appropriately for modification into UCINET-ready symmetric files. The researcher then made copies of the spreadsheet and converted the call information into binary form (0 = no tie or call, 1 = tie or call) and formatted appropriately for modification into UCINET-ready excel files. The files were then uploaded into UCINET and converted into UCINET files. A UCINET data file on the traditional social network structure was also generated. As noted previously, important attributes of the network that should be assessed to better understand the network include centrality, degree, and betweenness [14,18]. Therefore, analyses primarily focused on such attributes.

Using UCINET, each of the following analyses were run: (1) ego-network density to look at the size of the ego-network, ties (or degree), diameter, two-step reach, reach efficiency, brokerage and betweenness, (2) network degree centrality: Freeman’s approach and Bonacich’s approach, and (3) cliques to determine subgroups.

**Figure 2:**
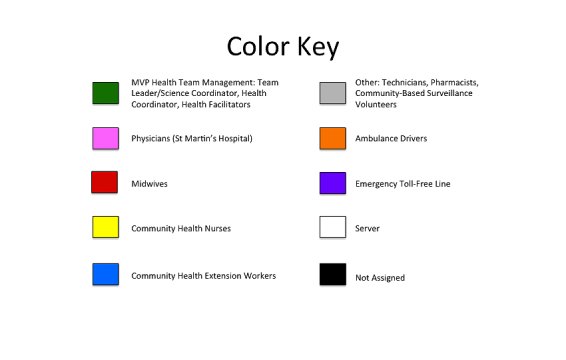
Color key for sociograms made in NetDraw on the traditional organizational structure and monthly Airtel closed user group call data.

To obtain a visual depiction of the network, sociograms were created using NetDraw. The UCINET data files were individually uploaded into NetDraw. To differentiate the actors in the mobile phone CUG by their role on the MVP Health Team, colors were assigned to the different cadres of health workers (see Figure 2). As networks can change over time, these network maps allowed for quick review of whether and how the network may have changed with time. From these sociograms, connectedness of the network was also noted.

Network data from interviews with 23 key informants, from the mobile phone CUG, were used as contextual and supplementary information to the analyses of the entire network. The interviews primarily focused on mobile phone use within and outside of the mobile phone CUG, in-person communication, and advice channels. Call journals that were kept by the same key informants as the interviews for at least a 2-week period were used in a similar manner to the interviews. The journals were used to document calls made or received, purpose of call, and whether those calls were with members of the CUG. Transcripts of the interview were coded and analyzed using NVivo9. The call journals were transcribed into soft copies and analyzed using Excel calculation features and NVivo9 (for the purpose of call section).

For the ego-network analysis, the 10 largest ego-network sizes and the accompanying actors for each of the months were noted in an Excel sheet and then compared across all of the months for which there were data. Ten was chosen as the cut-off of the largest ego-network sizes across all of the months as the ego-network sizes dropped drastically in magnitude, to as low as zero (or no connection), typically after the first dozen largest ego-network sizes were identified. Furthermore, this allowed for consistency across the entire time period. For March, April, July, and August 2011, there were 11 actors that were identified with the largest ego-network sizes. In these instances, there were at least 2 actors in the top 10 who had the same value for their ego-network size. Overall, the actors who had among the 10 largest ego-network sizes were designated as central actors. Interview and call journal information provided additional contextual information on these central actors, where possible.

A similar approach was taken for the socionetwork-level (or complete network-level) centrality analyses as similar trends in the data were observed. Both Bonacich and Freeman tests were run on the network data and the top 10 values were compared across each month. Bonacich represents influence and Freeman represents power. Analyses of cliques were conducted, as well. The clique analysis was set to look at subgroups with 3 or more actors, as the smallest health team unit includes one Community Health Nurse and two Community Health Extension Workers.

## Results

The results are discussed below. The monthly results are then followed by a comparison and overview of all the time periods, inclusive of the interview and call journal information.

### Facsimile of Traditional Network

**Figure 3:**
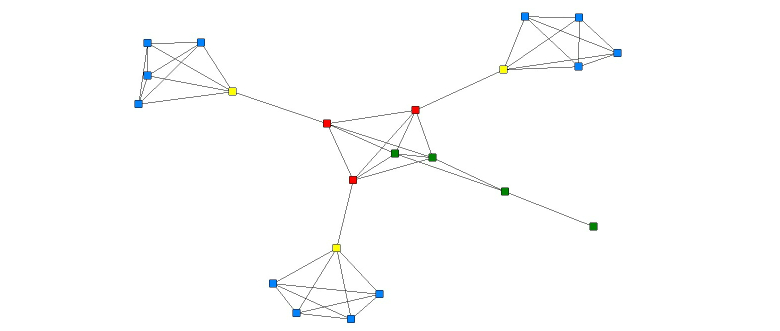
Sociogram of the traditional network where the relational tie is communication.

A facsimile of the traditional network was developed and analyzed. There were no disconnected actors in this network. According to the results of the analyses, Health Facilitators, midwives, and Community Health Nurses have the largest ego-networks. Midwives and Community Health Nurses have the highest values for the normalized broker measure. Based on the centrality measures, the midwives and Community Health Nurses have the most power (largest Freeman Betweenness Centrality measures,) and Community Health Nurses have intermediate influence in the network (Health Facilitators and midwives had the greatest influence/largest Bonacich values). A NetDraw image of the facsimile of the traditional network provides an illustration of the above results (see Figure 3).

### Mobile Phone Closed User Group Network

#### On Relational Ties and Disconnections

While the number of members of the mobile phone CUG is noted to be 79, the number does vary over time due to new hires and individuals leaving. In March 2011, the number of members in the CUG was 78. Over this month, 525 relational ties were made out of a possible 3081 ties. For the months of April 2011 to September 2011, the mobile phone CUG had 79 members. In April, the number of relational ties increased to 770. The number of relational ties increased in May to 856 and then decreased to 814 in June and July. The number of ties increased substantially to 1106 in August, but then decreased to 908 ties in September.

**Figure 4:**
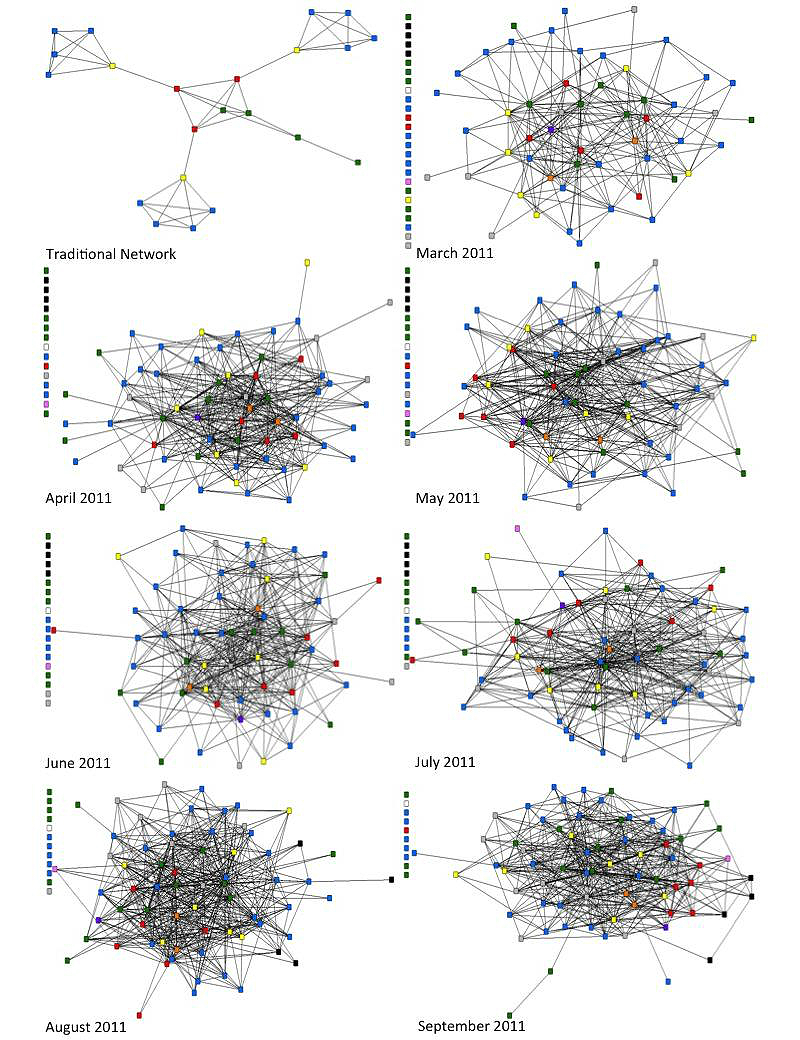
Depiction of sociograms from March 2011 through September 2011 juxtaposed with one another and the sociogram of the traditional network.

Over the months of March 2011 to September 2011, the number of actors having no relational ties decreased over time. In March, there were 23 actors disconnected from the network and in September, there were only 10 disconnected actors in the network (see Figure 4).

#### Identifying the Central Actors

There were several key actors that were consistently influential or central across all of the 7 months, based on their ego-network measures. All four were members of the Health Team Management. One individual, a Community Health Nurse, was a key central actor in all but one month. For Bonacich Centrality, 2 Community Health Extension Workers were highly prominent in 5 of the 7 months. A midwife, Community Health Nurse, and Community Health Extension Worker were identified as central actors in 4 of the 7 months. With the Freeman Betweenness Centrality, there were 5 individuals that were identified as central actors for 6 of the 7 months. They were 4 Health Team Management members and 1 ambulance driver. When taking the ego-network size, Freeman Betweenness Centrality and Bonacich Centrality measures into consideration, 2 Health Team Management members were identified as the most central actors (high values across all measures).

### Ego-Networks

Ego-network size, density, diameter, reach efficiency, and normalized brokerage values of ego-networks were calculated to help identify the most central actors in the mobile phone CUG network (see Table 1). Across the months, the largest ego-network size ranged from 24 to 40 with a median of 27. Density ranged from 43.5 to 52.8, indicating that, overall, the network is loosely connected and this was consistent over time. The largest diameter of an ego-network was in March 2011 (diameter size of 19.6). For the remaining months, this reduced to 4 or 5, with most months at 4. Across all 7 months, most of the actors had little (<50) to no reach efficiency. The highest normalized brokerage values ranged from 70% to 78%. Over the months, the general trend for the number of subgroups in the network consisting of 3 or more actors increased. The number of subgroups ranged from 92 in March to 423 in September. Among the health worker types, midwives tended to be clustered together in March, April, May, June, August, and September 2011.

**Table 1:** Summary of ego-network measures of the Airtel mobile phone closed user group by month from March 2011 to September 2011 (source: Airtel closed user group call data).

	March	April	May	June	July	August	September
Largest ENS^a^	24	42	41	41	38	45	40
Range of top 10 largest ENS	19–24	22–42	25–41	24–41	19–38	28–45	23–40
Largest density	43.5	52.8	48.7	47.7	46.8	52.7	43.9
Density range for 10 largest ENS	30–43.5	21.6–52.8	29.3–48.7	29–47.7	22.3–46.8	30.6–52.7	24.6–43.9
Largest diameter	19.6	4	4	5	4	4	4
# Actors with no RE^b^	23	16	19	18	15	12	10
# Actors with RE > 50	12	13	8	8	8	7	9
Highest NBV^c^	70%	78%	71%	71%	78%	71%	75%
NBV range for 10 largest ENS	59%–70%	54%–78%	51%–71%	52%–71%	53%–78%	47%–71%	56%–75%
# Subgroups w/ > 3 actors	92	225	313	273	284	380	423

^a^ENS–Ego-network size.

^b^RE–Reach efficiency.

^c^NBV–Normalized brokerage values.

### Centrality

The highest normalized Bonacich Centrality values increased from March through August and then decreased in September. The highest Bonacich Centrality values across the 7 months of network data ranged from 30.7 to 54.7. There was some overlap in the actors identified as central by their ego-network size and Bonacich Centrality measure. In March, there were 8 actors identified as central to the network based on their ego-network size and Bonacich Centrality. In June, July, and August, the number dropped to 3 actors.

There was more overlap of ego-network size and Freeman Betweenness Centrality than ego-network size and Bonacich Centrality. The range of the overlap across the months was small. The values ranged from 6 to 9 actors. Across all the months, the Freeman Betweenness Centrality was varied. This is evidenced by the range, mean, and standard deviation of the normalized values (see Table 2). Overall, the network centralization index was low, ranging from 5.3% in March to 11.01% in April, with a global median of 7.3% and global mean of 7.6%.

**Table 2:** Summary of Bonacich and Freeman centrality measures of the Airtel mobile phone closed user group by month from March 2011 to September 2011 (source: Airtel closed user group call data).

		March	April	May	June	July	August	September
**Freeman Betweenness Centrality (FBC)**								
	# Actors with highest FBC and ENS	9	8	7	6	8	7	8
	Range of normalized values	0–5.9	0–11.6	0–7.8	0–9.6	0–6.6	0–6.8	0–9.6
	Mean	0.68	0.7	0.65	0.67	0.82	0.8	0.95
	Standard deviation	1.3	1.7	1.2	1.3	1.4	1.4	1.6
	NCI^a^	5.3%	11.01%	7.3%	9.1%	5.8%	6.1%	8.8%
**Bonacich Centrality (BC)**								
	# Actors with highest BC and ENS	8	6	5	3	3	3	4
	Highest BC value	37	41	36.5	54.2	52	54.7	30.7

^a^ network centralization index

### Qualitative Findings

The qualitative findings corresponded with the social network analysis results. Individuals within the mobile phone CUG called other members of the CUG, whether they were within or outside of their catchment area. This was unlike the in-person communication and pre–mobile phone CUG communication that tended to be limited to intracatchment communication. In addition, the informants regularly established communication with the ambulance drivers in cases of emergencies.

## Discussion

We identified the central players in the mobile phone CUG network using both ego-network and sociocentric (or complete network) approaches. The ego-network size was the first indicator to be analyzed, as the size of the individual network is indicative of being a central figure in the network. Individuals with larger ego-networks may have greater influence in the network as compared to others with smaller ego-networks.

Based on the traditional network structure, Community Health Nurses form a bridge between the Community Health Extension Workers and the Health Team management (directly linking to the Health Facilitators). Community Health Nurses also connect the Community Health Extension Workers with midwives. Therefore, it was surmised that Community Health Nurses would be central actors in the network as they should have a large ego-network size and serve as intermediaries (high broker values). However, when analyzing the actual mobile phone CUG network data, this was found to not be the case.

Across all of the months, the ego-network diameters, even at their highest levels, were low. This indicates that the ego-networks were not very extensive. This may be due to most individuals in the network having set communication patterns and these communications taking place in a pre-defined subgroup (such as a catchment area). Similarly, the reach efficiencies of the ego-networks were low to moderate. Those with smaller ego-network size tended to have higher reach efficiencies. This could be due to the fact that those with smaller ego-networks made relational ties with those with larger ego-networks, while those with larger ego-network sizes had relational ties to those with small ego-networks (and thus, the extent of the network beyond the alters was limited). Accordingly, those with larger ego-networks tended to play the broker role relatively frequently. In the overall networks, those who did fulfill the role of a broker filled that role at a moderate to high frequency.

The socionetwork indicators allowed for additional information on key actors in the network. Since the Bonacich Centrality calculation was based on a positive beta, the resulting data were representative of the most central actors who hold power due to being connected to others in the network who are well connected. From the results, it appears that power (based on being connected to those who are well connected) is limited. The power lies with few individuals. This finding may help explain why there was less overlap between the ego-network size and Bonacich Centrality than the Freeman Betweenness Centrality. The Bonacich Centrality findings of having a network with limited power correlate with the ego-network size results. If the network were to have more power, those with larger ego-network sizes would be connected to others with larger ego-network sizes.

Looking at betweenness via the Freeman Betweenness Centrality measure, across all of the months, there was considerable variation in this measure. With that noted, it was not surprising to find that ambulance drivers were among the central actors in the network and had high levels of betweenness activity. Ambulance drivers receive calls from any of the health care workers and then may have to relay that information to others in the network or beyond. However, the overall network centralization index values for the Freeman Betweenness Centrality measures were low, meaning that connections in the network can be made without intermediaries. Despite the traditional network structure, the mobile phone CUG structure has disrupted this, allowing individuals who would not normally communicate with one another to make such connections directly, rather than going through other actors.

Subgroups, or cliques, exist in the network, as evidenced by the results from the clique analysis in UCINET. Therefore, the mobile phone CUG has a community structure. This community structure may partially explain the fact that the networks were found to be moderately connected. Some of the subgroups may be more tightly connected within their respective group and weakly connected with other subgroups. As most of the Health Team is divided into catchment areas, there may be natural divisions. There may also be natural divisions among the health worker types (eg, midwives communicate closely among one another). The mobile phones would also allow for additional nontraditional cliques to form. Further analyses on cliques and an additional measure for power, structural holes, would need to be carried out to better determine the community structure [23,24].

Limitations of these analyses include focusing primarily on the largest values for the measures. Also, 10 was an arbitrary cut-off number but allowed for interesting comparisons among the various measures and more manageable management of the results. In terms of context, there were individuals experiencing phone issues at this time period due to blocked SIMs over the time period of interest. The blocked SIM issue was resolved after September 2011. Therefore, those who were disconnected from the network may, in reality, may not truly be disconnected. Additionally, there may have been key players experiencing phone issues throughout the duration of the time period who were not labeled as such due to their limited phone connectivity.

### Conclusion

Based on the traditional network structure, it would appear that the Community Health Nurses would be highly prominent, or central, in the network. However, this was not the case, meaning that the evidence does not support Community Health Nurses being the most central actors in the network. Rather than Community Health Nurses, it was the members of the Health Team Management that were the more central players in the network. While this finding appears to support evidence that the traditional chain of command and communication flows have been “disrupted” by the mobile phone CUG, the Health Team management structure may, itself, be a confounder to the relationship between the mobile phone CUG and communication flows.

Nevertheless, it is evident that social network analysis methods can be a useful analytical tool, especially in the context of mHealth, health services, and operational/managerial research. The results on the voice traffic in the mobile phone CUG and the resulting social network structure can be used by the MVP Ghana Health Team to reflect on their current work-related mobile phone use and identify opportunities and areas to better manage team communication. For example, with the Health Team Management being such central actors within the network, they may be receiving multiple calls on the same topic or calls that could be better addressed by another actor in the network. Therefore, policies could be put into effect that could divert such calls to the appropriate channels and free up this time for the Health Team Management to attend to other tasks. The Health Team could also conduct similar social network analyses at later time periods to follow-up on any changes that they may put into place regarding the use of the mobile phone CUG.

Therefore, the social network analysis methodology can be used to determine the most conducive/productive structure for an organization/team, identify gaps in communication, identify key or central actors with greatest influence, and more. In the future, health outcomes research should also attempt to incorporate social network analysis methodology, as organizational structure of health service teams, organizations, and even communities may have an impact on the health outcomes of the communities being served.

## Abbreviations

BC: Bonacich centrality
CUG: closed user group
ENS: ego-network size
FBC: Freeman betweenness centrality
mHealth: mobile health
MVP: Millennium Villages Project
NBV: normalized brokerage values
NCI: network centralization index
RE: reach efficiency
